# Effect of Cocoa Supplementation on the Biochemical and Clinical Profile and the Somatosensory Processing of Diabetic Peripheral and Autonomic Neuropathy: A Randomized Clinical Trial

**DOI:** 10.3390/ijms26168033

**Published:** 2025-08-20

**Authors:** Rebeca Kababie-Ameo, Gabriela Gutiérrez-Salmeán, Luisa Fernanda Salinas-Hernández, Virgilio Eduardo Trujillo-Condes, Israel Ramírez-Sánchez, Carlos A. Cuellar

**Affiliations:** 1 Centro de Investigación en Ciencias de la Salud, Facultad de Ciencias de la Salud, Universidad Anáhuac México, Huixquilucan 52786, Estado de Mexico, Mexico; rebeca.kababie@anahuac.mx (R.K.-A.); gabrielasalmean@yahoo.com (G.G.-S.); 2 Escuela Militar de Graduados de Sanidad. Secretaría de la Defensa Nacional, Miguel Hidalgo 11200, Ciudad de Mexico, Mexico; 3 Facultad de Ciencias de la Salud, Universidad Anáhuac México, Huixquilucan 52786, Estado de Mexico, Mexico; luisafsh438@gmail.com; 4 Facultad de Medicina, Universidad Autónoma del Estado de México, Toluca 50180, Estado de Mexico, Mexico; vetrujilloc@uaemex.mx; 5 Sección de Estudios de Posgrado e Investigación, Escuela Superior de Medicina del Instituto Politécnico Nacional, Miguel Hidalgo 11340, Ciudad de Mexico, Mexico; israel.ramirez14@hotmail.com; 6 Escuela de Ciencias del Deporte, Universidad Anáhuac México, Huixquilucan 52786, Estado de Mexico, Mexico

**Keywords:** cocoa, polyphenols, diabetic neuropathy, type 2 diabetes, diabetes mellitus, antioxidants, cardiometabolic risk factor, H-reflex, somatosensory processing

## Abstract

Peripheral and autonomic neuropathy are common in type 2 diabetes; they are associated with oxidative stress and inflammation. Cocoa, rich in polyphenols, may offer neuroprotective benefits. This study evaluated the effect of cocoa supplementation on the biochemical, clinical, and somatosensory profile of neuropathy in individuals with type 2 diabetes. A 12-week, double-blind controlled trial involved 39 subjects randomized to receive cocoa capsules (50 mg polyphenols) or placebo (methylcellulose). Evaluations included glycemic and lipid profiles, neutrophil/lymphocyte ratio, blood pressure, standardized questionnaires, anthropometric measurements, and the rate-dependent depression of the H-reflex. In the cocoa group, the Toronto score decreased by 2.63 points and the BEST score decreased by 1.45 points. In the placebo group, these reductions were 1.84 and 2.21 points, respectively. Neither difference was statistically significant between groups (*p* > 0.05). Quality-of-Life questionnaire score decreased by 9.2 points in the cocoa group, but without significant difference to the placebo group (*p* = 0.501). Fasting glucose and HbA1c levels decreased in the placebo group by 38 mg/dL (0.28%) but were not significantly different from the cocoa group (*p* > 0.05). No other intra- or inter-group differences were significant (*p* > 0.05). Cocoa supplementation did not show significant improvements over the placebo in the measured outcomes. Both groups showed persistent abnormalities in spinal somatosensory processing, with an RDD of the H-reflex ≥ 0.5.

## 1. Introduction

Type 2 diabetes (T2D) is a metabolic disease characterized by the progressive loss of insulin secretion with different grades of insulin resistance and is considered the most common type of diabetes, representing more than 90% of all cases [1].

Hyperglycemia is the primary factor linked to macrovascular and microvascular complications. The excess of the substrate triggers inflammation and oxidative stress, activating metabolic pathways that contribute to these complications, including diabetic neuropathy (DN) [2].

Diabetic neuropathy is defined as a group of disorders presenting signs and/or symptoms of nerve dysfunction with different clinical manifestations in diabetic peripheral neuropathy (DPN) and diabetic autonomic neuropathy (DAN) [3,4]. It has been reported that approximately 50% of people living with diabetes develop this condition [5,6]. Distal symmetric polyneuropathy or DSP is the most common clinical manifestation and it refers to the presence of signs or symptoms related to peripheral nerve dysfunction in people living with diabetes after the exclusion of other causes. Symptoms of DSP include allodynia, hyperalgesia, numbness, tingling, and pain, specifically in the hands and lower limbs [7]. Gastrointestinal DAN, on the other hand, refers to the dysfunction of the autonomic nervous system that controls the gastrointestinal tract. This condition can cause nausea, vomiting, early satiety, diarrhea, and constipation, significantly impacting the quality of life of those affected [8]. The mechanisms for developing DN are mainly oxidative stress [9] and inflammation [7]. Hyperglycemia can induce oxidative stress, stimulate macrophages to cytokine secretion, and provoke the glycation of structural and functional proteins producing advanced glycation end products (AGEs) [7,10]. AGEs can decrease axonal transport inside neurons, leading to their degeneration. In addition, hyperglycemia increases oxidative stress and inflammation, affects the structural integrity of neurons, and alters blood flow in the nervous system, therefore generating nerve dysfunction [11].

Some factors that increase the possibility of developing DN are the duration of diabetes, high glycated hemoglobin (HbA1c) values, smoking, obesity, high blood pressure, and dyslipidemia, all of which are metabolic risk factors and are associated with persistent inflammation and oxidative stress [9,12].

The Hoffman reflex (hereafter referred to as the H-reflex) is produced by an electrical pulse applied to a peripheral nerve and is recorded in a specific muscle, allowing for an evaluation of the nerve fibers and associated neural circuits under both normal and pathological conditions [13]. Recently, a test based on the Rate-Dependent Depression (RDD) of the H-reflex has been proposed to evaluate the somatosensory processing in the spinal cord. It refers to the decrease in the amplitude of the H-reflex during consecutive trains of electrical stimulation at frequencies >1 Hz in healthy subjects. Interestingly, the RDD is altered in type 1 diabetes and T2D in humans, which is attributed to the disinhibition of spinal circuits dependent on GABAergic modulation [14,15]. Therefore, it has been suggested that the RDD of the H-reflex could objectively and noninvasively detect alterations in relation to spinal somatosensory processing, being relevant in the assessment of DPN [16].

Currently, the management of DN involves lifestyle changes to control glycemia and associated risk factors, along with pharmacological treatment for pain relief, such as Pregabalin, Duloxetine, Gabapentin, and Amitriptyline. However, these drugs can cause adverse effects, particularly in older adults. Additionally, their dosages may need to be restricted in patients with comorbidities such as cardiovascular or chronic kidney disease, potentially limiting their long-term efficacy [12,17,18].

Based on the above, the consumption of phenolic compounds such as flavonoids, which are present in various plant-based foods, has been proposed to mitigate symptoms associated with DN [19,20]. The benefits include better glycemic and lipid control, improved body composition, and reduced inflammation and oxidative stress markers—all without the adverse effects associated with medications [19,20].

Cocoa is rich in flavonoids, particularly flavanols like (-)-epicatechin and (+)-catechin, which have antioxidant properties [21]. Additionally, it contains around 300 components, including quercetin, naringenin, luteolin, apigenin, and tryptophan [22]. These phenolic compounds are also linked to the regulation of various parameters, including body weight, fat mass, lipid and glycemic profiles, insulin resistance, blood pressure, and inflammation markers—all of which play a role in the development of DN [23].

The consumption of catechins has been associated with a decrease in hyperglycemia and insulin resistance through the modulation of proinflammatory cytokines, the activation of signaling pathways that allow for the maintenance of adequate function of the mitochondrial respiratory chain, and a decrease in neuropathic pain through different mechanisms, one of the most important being the modulation of this inflammatory response [24,25]. Similarly, a preclinical study showed that the consumption of catechins can attenuate DAN by increasing antioxidant enzymes in the vagus nerve and decreasing other oxidative stress markers [26].

In in vitro studies, epicatechin reduced the macrophage secretion of Tumor Necrosis Factor alpha (TNFα) and monocyte chemoattractant protein-1 (MCP-1), as well as suppressing the production of Interleukin-6 (IL-6) and Interleukin-8 (IL-8) in stimulated whole blood cell cultures. In addition, cocoa and flavonoids reduced reactive oxygen species (ROS) generation in cells [25]. Furthermore, in intestinal epithelial cells, cocoa procyanidins have been reported to modulate the activation of Nuclear Factor-Kappa B (NF-κB) mediated by TNFα [25]. The flavonol quercetin has demonstrated anti-inflammatory, antioxidant, and analgesic actions, particularly in animal models, through mechanisms involving the nervous system [25,27]. On the other hand, studies like the COSMOS trial did not find a reduction in the risk of incident T2D with a cocoa extract supplement after a median follow-up of 3.5 years [28]. In addition, a study administering a beverage with low- and high-flavanol cocoa for 4 weeks reported no improvement in the indices of fasting insulin resistance [29].

Considering the above, this study aimed to evaluate the effect of cocoa supplementation on the biochemical parameters, clinical profile, and spinal sensorimotor processing of DPN and gastrointestinal DAN in subjects living with T2D.

## 2. Results

**Figure 1:**
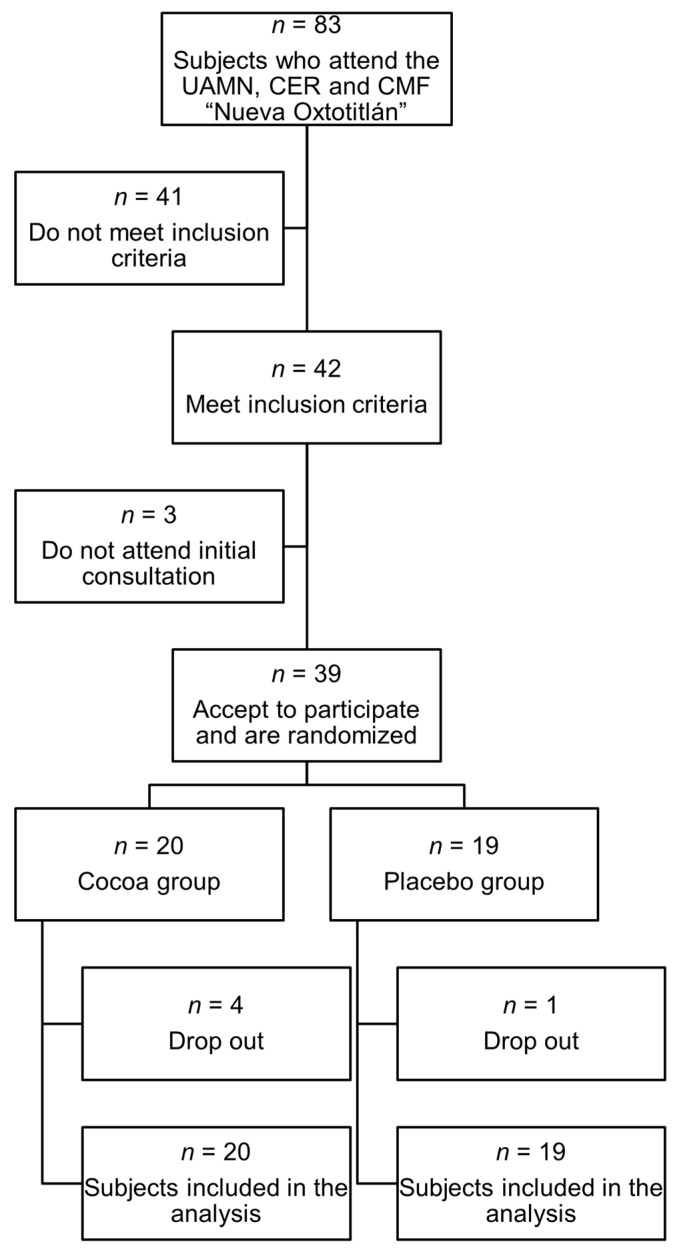
Flow diagram for study participants.

A total of 39 subjects were recruited, 19 of whom were randomized to the control group and 20 to the intervention group, while 34 completed the study. The recruitment flow chart is presented in Figure 1.

Of the 39 subjects who entered the study, 56.4% were women and 43.6% were men (mean age of 53.7 ± 7.52 years), with a duration of T2D diagnosis of 12 ± 8.3 years. At baseline, after comparing the cocoa and placebo groups, there were statistically significant differences in the parameters of weight (73.5 ± 14.1 vs. 82.8 ± 13.8; *p* = 0.047), body mass index (BMI) (28.7 ± 4.35 vs. 32 ± 5.32; *p* = 0.043), and Toronto Clinical Scoring System (TCSS) (9.31 ± 3.98 vs. 6.89 ± 3.47; *p* = 0.047), respectively. The demographic characteristics of subjects are detailed in Table 1.

### 2.1. Anthropometric Indicators

After 12 weeks, anthropometric variables remained stable throughout the intervention, with no statistically significant differences found either within or between groups (Table 2). The change from baseline in body weight was 0.38 ± 2.72 kg (*p* > 0.05) in the cocoa group and −0.42 ± 2.18 kg in the placebo group (*p* > 0.05), with a Cohen’s d of 0.38. Similarly, in relation to BMI, a difference was found of 0.12 ± 1 kg/m^2^ and −0.13 ± 0.84 kg/ m^2^ (*p* > 0.05), respectively, with a Cohen’s d of 0.27. Waist circumference remained without significant change, with a decrease of 0.8 ± 2.19 cm (*p* > 0.05) in the cocoa group and 1.38 ± 4.11 cm (*p* > 0.05) in the placebo group, leading to a lack of change in Waist-to-Height Ratio (WtH) ratio (−0.005 ± 0.01 vs. −0.009 ± 0.02; *p* > 0.05, respectively; Cohen’s d = 0.17). Abdominal circumference presented a slight decrease in both groups but without a statistical or clinical relevance, with a change of −0.75 ± 2.45 cm (*p* > 0.05) in the cocoa group and −1.02 ± 3.07 cm (*p* > 0.05) in the placebo group, with a Cohen’s d of 0.09.

### 2.2. Biochemical Indicators

**Figure 2:**
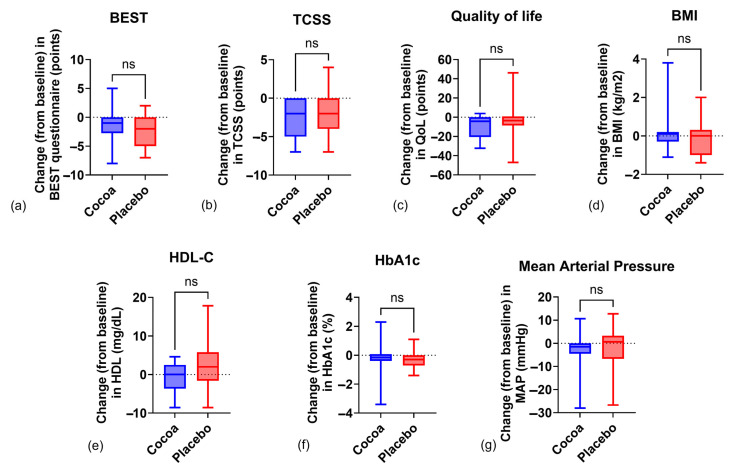
Change from baseline in different variables in the cocoa vs. placebo groups. (**a**) BEST questionnaire, (**b**) Toronto Clinical Scoring System, (**c**) Quality-of-Life questionnaire, (**d**) body mass index, (**e**) high-density lipoprotein cholesterol, (**f**) glycated hemoglobin, and (**g**) mean arterial pressure. ns: non-significant.

As shown in Table 2, statistically significant differences were found in blood glucose and HbA1c levels in the placebo group after intra-group analysis; however, when comparing both groups, no statistical difference was found (glucose: *p* = 0.33, Cohen’s d = 0.42; HbA1c: *p* = 0.58, Cohen’s d = 0.11). Regarding lipids, triglycerides (TGs) decreased without statistical significance, −4.96 ± 60.2 mg/dL (*p* > 0.05) in the cocoa group and −13.9 ± 44.6 mg/dL (*p* > 0.05) in the placebo group, with a Cohen’s d of 0.16. Meanwhile, high-density lipoprotein cholesterol (HDL-C) decreased by 0.86 ± 3.71 mg/dL (*p* > 0.05) in the cocoa group and increased by 2.18 ± 6.23 mg/dL (*p* > 0.05) in the placebo group (Table 2). When comparing both groups, no statistical difference was found (*p* = 0.070; Cohen’s = −0.59), as seen in Figure 2. Those changes led to the maintenance of the TG/HDL ratio (−0.02 ± 1.98 vs. −0.74 ± 2.13, cocoa vs. placebo groups, *p* = 0.55, Cohen’s d = 0.35), without statistical significance in the inter-group analysis (Table 2). Low-density lipoprotein cholesterol (LDL-C) had a slight increase in both groups but without achieving a statistically significant difference within or between groups, at 8.88 ± 22.1 mg/dL (*p* = 0.22) in the cocoa group and 4.15 ± 18.1 mg/dL (*p* = 0.33) in the placebo group. Changes in neutrophil/lymphocyte ratio were similar in both groups, and no statistical difference was found within or between groups (−0.09 ± 0.77 vs. 0.15 ± 0.45, *p* = 0.12, Cohen’s d = −0.38 cocoa vs. placebo group), as seen in Table 2.

### 2.3. Clinical Indicators

As shown in Table 2, there was a statistically significant difference in TCSS, both in the cocoa and the placebo group after 12 weeks, with a decrease of 2.63 ± 2.24 (*p* = 0.0001) and 1.84 ± 2.77 (*p* = 0.012), respectively. However, when comparing both groups, no statistical difference was found (*p* = 0.34, Cohen’s d = −0.31). A decrease in the Quality-of-Life score was observed in the cocoa vs. placebo groups (−9.19 ± 11.6, *p* = 0.0007 vs. −5.26 ± 19, *p* > 0.05, respectively), which implies an improvement in quality of life in the cocoa group. Regarding the BEST questionnaire, there was a statistically significant difference in both groups, where both decreased—cocoa: 1.45 ± 2.62 (*p* = 0.042) vs. placebo: 2.21 ± 2.59 (*p* = 0.015); however, when comparing groups, no statistical difference was found (*p* = 0.43, Cohen’s d = 0.29). Blood pressure showed a decrease in both groups—systolic (cocoa: 3.7 ± 11 mmHg vs. placebo: 3.78 ± 14.8 mmHg; *p* > 0.05), diastolic (cocoa: 2.32 ± 7.57 mmHg vs. placebo: 2.36 ± 10.1 mmHg; *p* > 0.05), and mean blood pressure (cocoa: 2.78 ± 8.02 mmHg vs. placebo: 2.84 ± 10.9 mmHg, *p* > 0.05). When comparing between groups, there was no statistical difference (*p* > 0.05). For the Bristol scale, a tendency towards the normalization of evacuations towards Bristol 3 and 4 is observed, specifically in the cocoa group, but when applying the Z Test, no statistical difference was found within or between groups (*p* > 0.05).

### 2.4. Sensorimotor Processing

The H-reflex was found in 16 subjects (41%)—8 in the cocoa group and 8 in the placebo group—at baseline, and in 10 subjects at their final evaluation for the placebo group. As seen in Table 2, when comparing the initial and the final means of the RDD of the H-reflex for each group, both remained above 0.5, which is considered as an indicator of dysfunction in somatosensory processing. At 0.1 Hz, RDD remained unchanged as expected; at baseline in the cocoa and placebo group, the values obtained were as follows: 1.09 ± 0.25 and 1.02 ± 0.12, *p* > 0.05, respectively. At the final evaluation, the values were as follows: 1.06 ± 0.23 and 1.03 ± 0.14, *p* > 0.05, respectively. For the frequency of 1 Hz, at baseline in the cocoa group, the RDD was 0.87 ± 0.35 and ended at 0.99 ± 0.28, *p* > 0.05, while in the placebo group, it was initiated at 0.88 ± 0.16 and at the final evaluation, this value was 0.89 ± 0.16, *p* > 0.05. At the 5 Hz frequency, the values of the RDD at baseline in the cocoa and placebo groups were 0.91 ± 0.67 and 0.71 ± 0.38, *p* > 0.05, respectively, and at the final evaluation were 0.89 ± 0.21 and 0.65 ± 0.27, *p* > 0.05, respectively. At the maximum frequency of stimulation (10 Hz), the value of the RDD in the cocoa group was 0.56 ± 0.47 and ended with 0.72 ± 0.25 *p* > 0.05, while in the placebo group, this value started at 0.71 ± 0.42 and at the final evaluation, it was 0.62 ± 0.30, *p* > 0.05.

## 3. Discussion

This randomized and double-blind clinical trial aimed to evaluate the effect of cocoa supplementation on the biochemical and clinical profile, as well as the spinal somatosensory processing, of diabetic peripheral and autonomic neuropathy in subjects living with type 2 diabetes.

Our study included anthropometric and biochemical tests, blood pressure measurement, application of questionnaires on quality of life (Diabetes 39) and gastrointestinal symptoms (BEST and Bristol), an evaluation of the RDD of the H reflex, and the TCSS instrument for the assessment of diabetic peripheral and gastrointestinal autonomic neuropathy in people living with T2D. Taken together, our study represents a comprehensive, though not exhaustive, approach to evaluate the effect of flavonoids on DN and its multisystem complications.

T2D is a metabolic disorder characterized by a state of persistent hyperglycemia that is associated with the development of complications such as DN [3]. In addition, factors that increase the possibility of presenting DN such as abdominal obesity, dyslipidemia, and arterial hypertension were identified in the participants [9,12].

According to the screening applied to 83 subjects, the presence of DN was detected in 50.6% of subjects living with T2D, which is similar to the value reported at local and global levels, estimating a prevalence of 50% [5,6].

When evaluating the anthropometric variables in the sample, it is highlighted that BMI persisted in the classification of overweight and obesity, as well as the abdominal and waist circumferences, which are indicators of adiposity and visceral fat accumulation; these were maintained above the recommendation in Mexico (less than 80 cm in women and less than 90 cm in men) [30,31]. The results are relevant since adipocytes of the visceral adipose tissue secrete, to a greater extent, inflammatory cytokines and ROS and are associated with the development of complications such as DN [32].

Regarding these parameters, the present study showed a similar result compared to the study of Simpson et al., who evaluated the effect of dairy-based beverages made with high-flavanol cocoa (609 mg of flavanols and 95 mg (-)-epicatechin per serving) or low-flavanol cocoa (13 mg of flavanols and 2 mg (-)-epicatechin per serving) for 4 weeks in pre-menopausal females who were overweight or with obesity grade I, reporting an increase of 0.29 kg in the high-flavanol cocoa group and a decrease of 0.86 kg in the low-flavanol cocoa group [29].

However, our study presented a smaller decrease in waist and abdominal circumference compared to that reported by other authors such as Dicks et al., who evaluated the effect of 2.5 g/day of flavanol-rich cocoa powder capsules for 12 weeks in subjects living with T2DM and hypertension, reporting a decrease in BMI of 0.4 kg/m^2^ (*p* > 0.05) and of 1.3 cm in waist circumference (*p* = 0.047) in the group receiving the cocoa powder [33]. A study by Munguía et al., reported that supplementation with a flavanol-enriched cocoa drink in overweight subjects for a period of 4 weeks improved anthropometric parameters such as weight (decrease of 2.4 kg; *p* < 0.05) and abdominal circumference (decrease of 3.5 cm; *p* < 0.05) [23]. Similarly, a study by León-Flores et al., found that the consumption of cookies enriched with 25 mg of epicatechin equivalents for 8 weeks decreased body weight by 3.2 kg (*p* < 0.0001), BMI by 1.3 kg/m^2^ (*p* < 0.0001), and waist circumference by 3.9 cm (*p* < 0.0001) in overweight subjects [34].

Discrepancies between the results of the present study and those reported in previous investigations may be attributed, among other factors, to (a) differences in polyphenol content across interventions—for example, cocoa powder capsules contained 207.5 mg of flavanols [33], the enriched cocoa drink provided 80 mg of flavonoids per serving, and cocoa-enriched cookies in [23], as well as the intervention in [34], used 25 mg of epicatechin equivalents, whereas the present study administered 50 mg of total polyphenols per day; (b) variations in sample characteristics, such as sex distribution and the presence of T2DM; and (c) differences in cocoa presentation, including capsules, cookies, and drinks.

The WtH ratio has been considered to be a sensitive indicator of cardiometabolic risk and it has been observed that the higher the index, the greater the risk of developing DM, hypertension, cardiovascular disease, and dyslipidemias. In this sense, a cut-off point of 0.5 has been proposed for different ethnic groups [35]. In the present study, the subjects maintained a WtH ratio of 0.6, which is above the proposed cut-off point (Table 2).

Regarding biochemical parameters, the placebo group showed a statistically significant decrease in glycemia and HbA1c levels after the 12-week intervention; however, when comparing both groups, the means did not reach statistical significance (*p* > 0.05). The decrease in glycemia in the cocoa group was also relevant since it had a reduction of 17 mg/dL, which, despite not presenting statistical significance, clinically improved the glycemic control of the patients. Furthermore, it is important since hyperglycemia contributes to oxidative stress and the secretion of proinflammatory cytokines that perpetuate nerve damage [7,10]. High glucose levels also produce AGEs, which modify intracellular signaling pathways for the release of proinflammatory cytokines and ROS [7] and can decrease the axonal transport of neurons, leading to their degeneration [11]. Additionally, it is relevant that although all subjects were subjected to pharmacological management for T2D and some of them also for dyslipidemia, they were not under clinical control [1]. The above underlines the importance of maintaining constant evaluations of the different clinical parameters to control and, if possible, to reduce the indicators of future complications.

When compared with other studies, it is generally agreed that interventions with cocoa or products derived from it decrease blood glucose concentrations. For example, a study by Munguía et al., reported that blood glucose decreased by 13.4 mg/dL (*p* < 0.01) [23], while a study conducted by Rostami et al., reported a decrease in HbA1c levels of 0.14% (*p* = 0.025) in subjects living with T2D and hypertension who consumed 25 g of dark chocolate with 450 mg of polyphenols for 8 weeks. However, the decrease in blood glucose reported was 7.84 mg/dL (*p* = 0.027), which is less than what was found in the present study, as mentioned above [36]. In our study, we found a decrease in HbA1c levels of 0.1% (1.04 mmol/mol) in the cocoa group and 0.28% (2.94 mmol/mol) in the placebo group; however, in both cases, the average remained above the objective established by the ADA, which is 7% [37].

Regarding the lipid profile, TG values had a slight decrease within groups but did not reach statistical significance, nor was there a difference between the groups. The final mean of HDL-C in both groups did not have a statistically significant difference despite the increase of 2.2 mg/dL in the placebo group (Figure 2), and the mean barely reached 40 mg/dL, with recommended values being >40 mg/dL for men and >50 mg/dL for women [38]. Elevated values of the TG/HDL index increase cardiovascular risk and are associated with metabolic syndrome. In both groups, the mean TG/HDL was >2.75 in men and >1.65 in women, which are values proposed as cut-off point for identifying metabolic syndrome [39]. As mentioned above, despite receiving pharmacological management for dyslipidemia, the participants remained above the cut-off point, which implies a risk factor for cardiometabolic diseases.

The neutrophil/lymphocyte ratio has been proposed as a biomarker of alterations in the function of the immune system and has been suggested to be a predictor of cardiovascular events and mortality in the general population [40]. Despite having different cut-off points, it is suggested that the higher the ratio, the greater the risk of morbidity and mortality; in this case, the neutrophil/lymphocyte ratio decreased slightly in the cocoa group, although without reaching statistical significance, while in the placebo group it increases, also without statistically significant difference (Table 2).

The clinical data evaluated in our study are relevant since they are closely related to the quality of life of patients. TCSS assesses DN and includes an evaluation of symptoms, reflexes, and sensitivity according to anatomical regions, establishing three grades—no DN, 0 to 5 points; mild DN, 6 to 8 points; moderate DN, 9 to 11 points; and severe DN, 12–19 points. This has been shown to have a good correlation with electrophysiology studies [41]. In the present study, a decrease of 2.63 points on the scale was observed in the cocoa group, changing the severity from moderate to mild DN, while in the placebo group, the decrease was 1.84 points, which represents a change from mild DN to no DN. In contrast to what was found in this work, a study that included subjects with DPN showed a decrease in TCSS of 5.72 points when supplemented with methylcobalamin + probucol, i.e., the combination of the active form of vitamin B12 and a lipid-lowering drug, which work together to improve the markers of oxidative stress such as malondialdehyde, superoxide dismutase, and glutathione peroxidase [42].

On the other hand, for the evaluation of quality of life, the “Diabetes 39” questionnaire was applied. This instrument contains 39 items that are grouped into sections of energy and mobility, diabetes control, anxiety and worry, social burden, and sexual functioning. The perception of quality of life is rated on a scale of 0 to 100, with 0 being a minimal affectation and 100 being an extremely serious affectation [43]. Based on our results, the decrease in the score in the cocoa group, after the intra-group analysis, reached a statistical significance and is translated as an improvement in the quality of life, which is of vital importance for the patient since it increases well-being and is one of the primary objectives of health professionals. The above could be associated with the decrease in the TCSS instrument score, as mentioned in the previous paragraph and shown in Table 2 and Figure 2.

Similarly to what was found in the present work, Munguía et al., reported an improvement in the quality of life, as measured with the EuroQol-5D (EQ-5D) questionnaire, after supplementation with a cocoa drink enriched with flavanols for 4 weeks [23]. The improvement could be associated with the conversion of tryptophan from cocoa to serotonin, which is a neurotransmitter related to mood [22].

In the case of blood pressure, systolic, diastolic, and mean arterial pressure were evaluated, and it was observed that they decreased, although not significantly, in both groups. These changes may be beneficial since high blood pressure is considered a risk factor for the development of DN, while the decrease in MAP implies a benefit at the level of cardiovascular risk [36,44]. Contrary to what was observed in the present study, Rostami et al., reported a decrease of 6.4 mmHg (*p* = 0.001) in systolic pressure with a dose of 25 g of dark chocolate for 8 weeks [36]. But, on the other hand, the study by Dicks et al., did not report changes in diastolic blood pressure and showed a decrease of 0.6 mmHg (*p* > 0.05) in systolic blood pressure in the group that received cocoa [33]. Mastroiacovo et al., reported that in subjects aged 62 to 79 years, SBP decreased by 1.6 mmHg (*p* = 0.14) and DBP decreased by 1.57 mmHg (*p* = 0.016) after consuming a beverage containing 48 mg of flavanols for 8 weeks [45].

GAN was evaluated using the BEST questionnaire and the Bristol scale. An improvement in the gastrointestinal symptoms of both groups was observed, reporting a decrease of 1.45 points for the cocoa group and 2.21 points for the placebo group between weeks 0 and 12 (Table 2 and Figure 2). Regarding the Bristol scale, no statistically significant difference was found when comparing cocoa and placebo groups, but it can be observed that there is a slight improvement in the consistency of the evacuations to Bristol 3 and 4 throughout the 12-week intervention, particularly in the cocoa group (Table 2). These changes could be related to the small contribution of fiber given through supplementation with both cocoa and methylcellulose in the placebo, which is soluble fiber.

Finally, for the sensorimotor evaluation using the RDD of the H-reflex, the amplitude of the evoked potentials was determined for each electrical pulse and at all stimulation frequencies; the ratio of the amplitude of the Hn … /H1 pulses was determined to establish the modulation of spinal excitability. A ratio ≥ 0.5 for any stimulation frequency was considered as an indicator of dysfunction in somatosensory processing according to Marshall et al. [14].

According to a report by Worthington et al., in 18.7% of subjects living with diabetes, with a higher proportion of subjects with T2D vs. T1D, who made up their sample, the H-reflex was absent [16,46], while as described in the study by Salinas et al., the H-reflex was absent in 36% of subjects with T2D [15]. On average, it is estimated that the H-reflex may be absent in 40% of patients with diabetes, especially among older subjects [47]. In our study, it was observed that the H-reflex was absent in 51.2% of the subjects. This may be attributed to several factors, including the severity of DN, as higher Michigan Neuropathy Screening Instrument scores are associated with a lower likelihood of detecting the H-reflex, as well as elevated HbA1c levels [15]. In this regard, it is unknown whether other complications of DM and the presence of comorbidities contribute to the loss of RDD.

A study by Marshall et al., reported that the RDD of the H-reflex is a test that could be used to differentiate subjects with painful and non-painful DN and to determine the response to drugs or compounds (i.e., supplements) that may reverse DN, as evaluated according to the somatosensory processing in the spinal cord [48]. It also showed that the alteration of the RDD is associated with greater burning pain, a high pain sensitivity, and relative heat hyperalgesia, which may lead to future research to work directly on these specific mechanisms and make the approach to these patients more effective [48].

The RDD of the H-reflex did not change throughout the study, since at the end of the intervention, the RDD was greater than 50% and had not reached the expected depression even at the maximum stimulation of 10 Hz, and an increase in the amplitude of the H-reflex wave was found, which, according to what was suggested by previous reports, is an indicator of alteration in the spinal inhibitory function, which is related to DN [14].

One possible explanation for the lack of statistically significant differences between the cocoa and placebo groups is that the total polyphenol dose in the supplement—particularly epicatechin—and the supplementation duration may not have been sufficient to produce the expected changes in DPN evaluation, as measured by the TCSS and RDD of the H-reflex. Moreover, the way in which the supplement was administered, i.e., capsules versus cookies or beverages, could also influence the effect, since exposing the bioactive elements of cocoa to heat or combining them with other elements that increase their bioavailability could be a relevant factor when evaluating their effectiveness.

Other factors include the presence of comorbidities in the subjects and the severity of the symptoms, since as there is greater affectation at the metabolic level and in the nervous system, the effect of the proposed management may not be sufficient. In addition, it could be suggested that although the dietary prescription was made for all subjects, some of them had a closer follow-up by their private physicians, while other subjects only had the follow-up given during the study; therefore, it could have impacted on the adherence to the eating plan and, secondarily, the effect of the supplementation.

The sample size in our study (*n* = 39) falls within the range reported in previous studies (15 to 60 participants) [23,29,33,34,36]. Since our sample size was calculated a priori, the risk of a Type II error was minimized; however, non-significant results should still be interpreted with caution.

DN is one of the main alterations related to DM, which still has several limitations in its diagnosis since the tests and instruments used depend on the symptoms reported by the patients. To date, there are no clinical trials that have assessed the effect of cocoa on DN including anthropometric, biochemical, clinical, and sensorimotor processing parameters. In this sense, our study is novel and integrates important aspects of the clinical manifestations of DN.

Finally, therapeutic options for DN are limited since they are palliative and focused on pain management; therefore, worldwide, this type of research takes on even more relevance given the high prevalence of DM and the high risk of morbidity and the deterioration in quality of life.

The results of this study highlight the homogeneity of the sample and that the effect of cocoa supplementation (at a dose of polyphenols of 50 mg per day for a duration of 12 weeks) did not show significant improvements in most of the parameters compared to the placebo group. It should be noted that both groups received a diet plan and follow-up for 12 weeks by those responsible for the study.

## 4. Materials and Methods

To investigate the impact of cocoa supplementation on diabetic peripheral and autonomic neuropathy in subjects living with T2D and DPN, a 12-week double-blind, randomized controlled clinical study was conducted between June 2021 and July 2024 in three centers located in the State of Mexico, Mexico: Universidad Anáhuac México; the Kidney Specialty Center; and the Family Medicine Clinic “Nueva Oxtotitlán”. The protocol was approved by the Faculty of Health Sciences Research Committee of the Universidad Anáhuac México (ID 202094) and was registered in ClinicalTrials (NCT05247034).

Subjects were randomized (using a table of random numbers previously generated in a spreadsheet by a person not involved in taking measurements and administering questionnaires) and allocated to the intervention group (diet + cocoa capsules, Cacao&Health^®^) or control group (diet + methylcellulose capsules). Each subject was followed for 12 weeks, with four appointments—at the beginning (baseline evaluation), in weeks 4 and 8 (check-up appointments), and at 12 weeks (final evaluation).

Subjects who had a score ≥ 2 on the Michigan Neuropathy Screening Instrument (MNSI) and signed the informed consent were enrolled. Exclusion criteria included subjects with known sensitivity or hyperreactivity to cocoa, pregnant women, subjects with previous neurological diseases, with implanted electronic devices (such as pacemakers) or use of orthopedic equipment (such as prostheses or splints), class C2-C6 venous insufficiency and/or with peripheral nerve damage in the lower limbs due to trauma, and subjects who modified the type of pharmacological treatment for glycemic control during the study.

### 4.1. Sample Size

The sample size was calculated using the formula for the difference in means, taking a previous study in subjects with DPN as a reference; the TCSS was used to assess the effect of the intervention [42]. The following parameters were considered from the previously mentioned study: a proposed mean difference of 0.776, representing the expected difference compared to the control group; a baseline standard deviation of 0.84; a Zα value of 1.96, corresponding to a two-tailed significance level of 95%; and a Zβ value corresponding to a statistical power of 80%. Based on these parameters, a total of 20 subjects per group was determined.

### 4.2. Dietary Intervention

All participants were given a personalized diet plan, calculated by the Mifflin-St. Jeor energy expenditure prediction formula [49] for subjects with obesity. Macronutrient distribution was made according to the Joslin Diabetes Center, as follows: 40–45% carbohydrates, 30–40% lipids, and 20–30% protein [50]. At each appointment, subjects were given two bottles with 60 capsules of 500 mg each (cocoa or placebo), which were returned empty to the researcher on the next visit. Four capsules were prescribed (two capsules in the morning and two capsules in the evening) for both groups—cocoa (50 mg of polyphenols per day) and methylcellulose for the placebo group. The polyphenol dose used in this study was selected based on a previous study [34] that utilized a similar product containing 25 mg of epicatechin equivalents. In that study, after eight weeks of supplementation, statistically significant differences were observed between the intervention and placebo groups in glucose levels, triglycerides, and the triglyceride-to-HDL ratio. The capsules and bottles of both groups had the same characteristics (size, shape, and color) to prevent identification by the participants. Cocoa capsules were manufactured by Cacao&Health^®^ (Baja California, Mexico), made with dried and ground cocoa beans, with 12.5 mg of polyphenols each.

### 4.3. Anthropometric Measurements

Weight, waist, and abdominal circumference were determined according to the Lohman technique [28]. Height was measured with a BSM 170 InBody digital stadiometer (InBody, Seoul, Republic of Korea). The WtH ratio was obtained by dividing waist circumference by the height, both in centimeters.

### 4.4. Clinical Parameters

Blood pressure was measured using a sphygmomanometer (Nebucor, Cuautitlan Izcall, Mexico, model HL-888JA), according to the Clinical Practice Guide for the Diagnosis and Treatment of Arterial Hypertension [51]. These evaluations were conducted at each appointment throughout the 12-week intervention. The TCSS, BEST questionnaire, Bristol scale, and Diabetes 39 Quality-of-Life questionnaire [43] were administered at both the beginning and end of the study.

### 4.5. Biochemical Parameters

Fasting triglycerides (TGs), high-density lipoprotein cholesterol (HDL-C), low-density lipoprotein cholesterol (LDL-C), glucose, HbA1c, neutrophils, and lymphocyte levels were obtained for each participant. The TG/HDL ratio was determined by dividing the serum triglyceride concentration by the serum HDL-C concentration, while the neutrophil/lymphocyte ratio was obtained by dividing the absolute neutrophil concentration by the absolute lymphocyte concentration [40].

### 4.6. Sensorimotor Processing: RDD of the H-Reflex

The RDD of the H-reflex test was performed through a bar electrode (MFI Medical, San Diego, CA, USA) connected to a bipolar constant current electrical stimulator (Digitimer DS8R, Digitimer, Hertfordshire, UK). The motor responses were recorded using surface electrodes (3M) connected to the signal acquisition and amplification system (LabChart and PowerLab 8/35, ADInstruments, Sydney, Australia). The signals were sampled at 10 kHz (band-pass filter of 0.5–500 Hz) and were stored on a computer for offline analysis. Once the recording and stimulation sites were identified, the subject’s skin was cleaned with an isopropyl alcohol pad. Micropore^®^ adhesive tape (Micropore, Elkton, MD, USA) was used to improve contact of the electrodes with the subjects’ skin. The electrodes for recording the H-reflex were placed as follows: the anode at the level of the Achilles tendon and the cathode above the inverted “V” between the calf muscles (gastrocnemius). The reference electrode was then placed 4 cm above the cathode. The electric pulse was applied to the skin behind the knee (popliteal fossa). First, the electrical stimulus (square pulse, 1 ms duration) was delivered every 10 s. The test started with an intensity of 0 mV, and pulses were given every 0.5 mV until the H-reflex (latency between 30 and 45 ms) exhibited a plateau or its amplitude decreased after two consecutive pulses. If the electrical pulse caused discomfort at any stimulus intensity, the test was terminated. Then, stimulus intensity vs. amplitude plots were created to identify the stimulus intensity at which the H-wave reached 40% of its maximum amplitude. This intensity was then applied for the RDD of the H-reflex protocol. Subsequently, 10 pulses were applied at 0.1 Hz as a control of the amplitude of the H-reflex, followed by three trains of five pulses each at stimulation frequencies of 1, 5, and 10 Hz. The interval between each pulse train was at least 30 s. The electrophysiological recordings were analyzed with Clampfit 10.0 software. The ratio of the amplitude of the H2 … H10/H1 pulses for 0.1 Hz, as well as of H2 … H5/H1 for 1, 5, and 10 Hz, were determined and averaged to establish the RDD of the H-reflex. A ratio ≥ 0.5 at stimulation frequencies ≥ 1 Hz was considered to be an indicator of the dysfunction in spinal somatosensory processing [14].

### 4.7. Statistical Analysis

The Shapiro–Wilk test was used to assess the normality of the data. Dependent and quantitative variables were expressed as mean and standard deviation, while categorical data such as sex were expressed as frequencies. Inferential analysis was performed by intention to treat; therefore, if a measurement was missing, the value of the immediately preceding measurement was repeated for each subject. For intragroup analysis, the paired T test or Wilcoxon test and ANOVA for repeated measures with Tukey’s post hoc test or Friedman tests with Dunn’s post hoc test were used, as appropriate. For the Bristol scale, the Z test was applied.

The unpaired T test or U Mann–Whitney test was performed for the intergroup analysis according to data distribution. Similarly, for the Bristol scale, the Z test was applied. A *p* < 0.05 was considered statistically significant. GraphPad Prism (GraphPad Software Inc., La Jolla, CA, USA) version 10.4 was used for statistics and graphical representation.

## Figures and Tables

**Table 1:** Baseline characteristics of the study (*n* = 39).

Variable	Cocoa (Mean ± SD)	Placebo (Mean ± SD)	*p*
Total (*n*)	20	19	
Age	53.6 ± 8.67	53.7 ± 6.34	ns
Sex M/F	10/10	7/12	
Weight (kg)	73.5 ± 14.1	82.8 ± 13.8	0.047
BMI (kg/m^2^)	28.7 ± 4.35	32 ± 5.32	0.043
Waist Circ (cm)	96.7 ± 7.49	103.5 ± 13.1	ns
Abd Circ (cm)	100 ± 9.31	106 ± 11.4	ns
SBP (mmHg)	126 ± 14.6	127 ± 15.5	ns
DBP (mmHg)	79.9 ± 8.23	78.1 ± 7.98	ns
BEST	9.35 ± 2.75	9.05 ± 2.77	ns
Bristol	3.65 ± 1.3	3.84 ± 1.5	ns
Altered Bristol (%)	40	57	ns
TCSS	9.31 ± 3.98	6.89 ± 3.47	0.047
QoL	43.4 ± 26.8	35.6 ± 22.6	ns
R2/R1 (*n* = 8)	0.79 ± 0.45	0.77 ± 0.32	ns
RDD 0.1 Hz	1.09 ± 0.25	1.02 ± 0.12	ns
RDD 1 Hz	0.87 ± 0.35	0.88 ± 0.33	ns
RDD 5 Hz	0.91 ± 0.67	0.71 ± 0.38	ns
RDD 10 Hz	0.56 ± 0.47	0.71 ± 0.42	ns
Glycemia (mg/dL)	169 ± 62.6	195 ± 85.7	ns
HbA1c (%)	7.82 ± 1.9	8.28 ± 2.02	ns
HbA1c (mmol/mol)	62 ± 20.7	66.9 ± 22.1	ns
TG (mg/dL)	182 ± 81.4	179 ± 58.8	ns
HDL (mg/dL)	37.8 ± 9.52	38.2 ± 9.12	ns
TG/HDL	5.12 ± 2.84	4.99 ± 2.13	ns
LDL (mg/dL)	112 ± 31.8	108 ± 31.3	ns
Neutrophil/lymphocyte	1.96 ± 0.86	1.76 ± 0.55	ns

Abd Circ: abdominal circumference; Waist Circ: waist circumference; HbA1c: glycated hemoglobin; HDL: high-density lipoprotein; BMI: body mass index; LDL: low-density lipoprotein; DBP: diastolic blood pressure; SBP: systolic blood pressure; QoL: quality of life; RDD: rate-dependent depression; SD: standard deviation; TCSS: Toronto Clinical Scoring System; TG: triglyceridemia.

**Table 2:** Intra-group and inter-group analysis of different variables throughout the 12-week intervention.

Variable (Mean ± SD)	Cocoa					Placebo				
	Week 0	Week 4	Week 8	Week 12	*p*	Week 0	Week 4	Week 8	Week 12	*p*
Weight (kg)	73.5 ± 14.1	74.4 ± 14.8	74.1 ± 14.7	73.8 ± 14.6	ns	82.8 ± 13.8	82.5 ± 13.9	83 ± 13.7	82.4 ± 13.9	ns
Change from baseline	0.38 ± 2.72					−0.42 ± 2.18 ⇞				
Waist Circ (cm)	96.7 ± 7.49	96.5 ± 7.08	96 ± 7.33	95.9 ± 7.28	ns	103 ± 13.1	103 ± 11.7	103 ± 12.0	102 ± 12.0	ns
Change from baseline	−0.80 ± 2.19					−1.38 ± 4.11 ⇞				
WtH ratio	0.6 ± 0.04	0.6 ± 0.04	0.6 ± 0.04	0.6 ± 0.04	ns	0.64 ± 0.09	0.64 ± 0.08	0.64 ± 0.09	0.63 ± 0.09	ns
Change from baseline	−0.00 ± 0.01					−0.00 ± 0.02 ⇞				
Glycemia (mg/dL)	169 ± 62.6			152 ± 58.4	ns	195 ± 85.7			157 ± 63.6	0.002 ¥
Change from baseline	−17 ± 42.8					−38.1 ± 55.9 ⇞				
HbA1c (%)	7.82 ± 1.9			7.72 ± 1.5	ns	8.28 ± 2.02			8 ± 2.04	0.04 ¥
Change from baseline	−0.17 ± 1.09					−0.27 ± 0.60 ⇞				
HbA1c (mmol/mol)	62 ± 20.7			60.9 ± 16.4	ns	66.9 ± 22.1			64 ± 22.3	ns
Change from baseline	−1.04 ± 11.9					−2.94 ± 6.58 ⇞				
TG/HDL	5.12 ± 2.84			5.1 ± 2.9	ns	4.99 ± 2.13			4.25 ± 1.31	ns
Change from baseline	−0.02 ± 1.98					−0.74 ± 2.13 ⇞				
HDL-C	37.8 ± 9.52			36.9 ± 7.5	ns	38.2 ± 9.12			40.4 ± 6.26	ns
Change from baseline	−0.86 ± 3.71					2.18 ± 6.23 ⇞				
Neutro/lymph	1.96 ± 0.86			1.87 ± 1.07	ns	1.76 ± 0.55			1.92 ± 0.61	ns
Change from baseline	−0.09 ± 0.77					0.15 ± 0.45 ⇞				
TCSS	9.31 ± 3.98			6.68 ± 4.41	0.0001 ¥	6.89 ± 3.47			5.05 ± 2.91	0.012 ¥
Change from baseline	−2.63 ± 2.24					−1.84 ± 2.77 ⇞				
QoL	43.4 ± 26.8			34.2 ± 25.8	0.0007 ¥	35.6 ± 22.6			30.4 ± 24.5	ns
Change from baseline	−9.19 ± 11.6					−5.26 ± 19 ⇞				
MBP (mmHg)	95.1 ± 9.57	94 ± 9.36	93.8 ± 8.35	92.3 ± 10.3	ns	94.2 ± 8.86	96.1 ± 9.39	92.9 ± 7.06	91.9 ± 7.69	ns
Change from baseline	−2.78 ± 8.02					−2.84 ± 10.9 ⇞				
BEST	9.35 ± 2.75	9 ± 3.12	8.05 ± 2.64	7.9 ± 2.67	0.042 ¥	9.05 ± 2.77	7.94 ± 1.87	7.42 ± 2.52	6.84 ± 2.6	0.015 ¥
Change from baseline	−1.45 ± 2.62					−2.21 ± 2.59 ⇞				
R2/R1	0.79 ± 0.45 (*n* = 8)			0.87 ± 0.20 (*n* = 8)	ns	0.77 ± 0.32 (*n* = 8)			0.72 ± 0.22 (*n* = 10)	ns
Change from baseline	0.06 ± 0.32					−0.02 ± 0.22 ⇞				
Bristol (% of alteration)	40			30	ns	57.8			52.6	ns
Change from baseline	−10					−5.2 ⇞				

Waist Circ: waist circumference; WtH ratio: waist-to-height ratio; HbA1c: glycated hemoglobin; MBP: mean blood pressure; Neutro/lymph: neutrophil/lymphocyte ratio; SD: standard deviation. ⇞: No statistically significant difference after intergroup comparison. ¥: statistically significant difference after intragroup comparison. ns: No statistically significant difference after intragroup comparison.

## Data Availability

Data are made available upon reasonable request by contacting the corresponding author.

## Abbreviations

The following abbreviations are used in this manuscript:

AGEs	Advanced Glycation End Products
BMI	Body Mass Index
DAN	Diabetic Autonomic Neuropathy
DBP	Diastolic Blood Pressure
DN	Diabetic Neuropathy
DPN	Diabetic Peripheral Neuropathy
DSP	Distal symmetric polyneuropathy
HbA1c	Glycated Hemoglobin
H-reflex	Hoffman Reflex
HDL-C	High-density lipoprotein cholesterol
IL	Interleukin
LDL-C	Low-density lipoprotein cholesterol
MAP	Mean Arterial Pressure
MCP-1	Monocyte chemoattractant protein-1
NF-κB	Nuclear Factor-Kappa B
QoL	Quality of Life
RDD	Rate-Dependent Depression
ROS	Reactive Oxygen Species
SBP	Systolic Blood Pressure
SD	Standard Deviation
T2D	Type 2 Diabetes
TCSS	Toronto Clinical Scoring System
TGs	Triglycerides
TNFα	Tumoral Necrosis Factor Alpha
WtH ratio	Waist-to-Height Ratio
